# Supplementary figures and images for: Polyarginine Cell-Penetrating Peptides Bind and Inhibit SERCA2
Source: Cells. 2023 Sep 26;12(19):2358. doi: 10.3390/cells12192358 (PMC10571751; doi:10.3390/cells12192358)

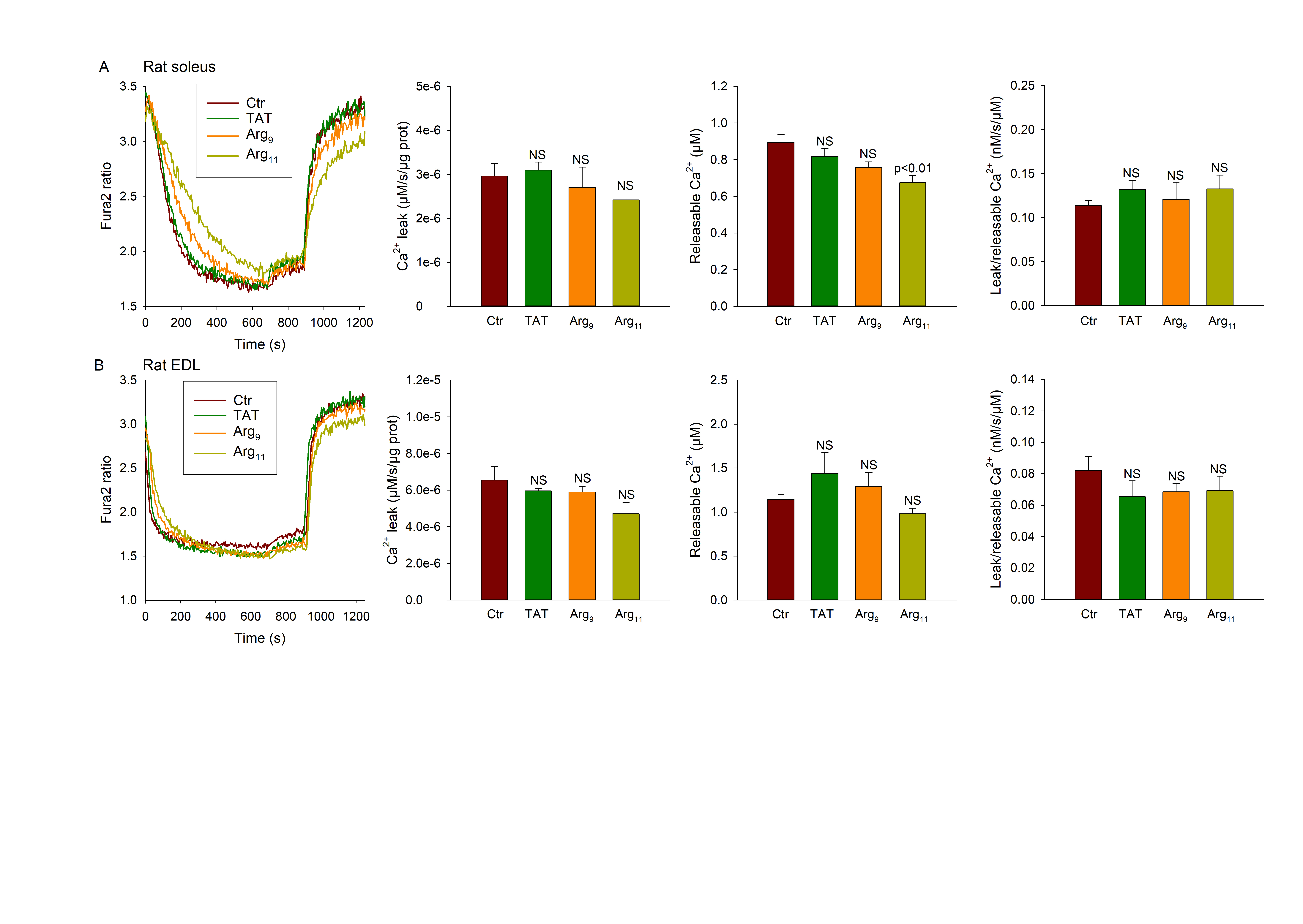

Supplement: Supplementary file 1 [file cells-12-02358-s001.zip › cells-2586455 Figure S1.JPG]
